# Comprehensive assessment of association between TLR4 gene polymorphisms and cancer risk: a systematic meta-analysis

**DOI:** 10.18632/oncotarget.21543

**Published:** 2017-10-06

**Authors:** Lu Ding, Qifeng Jiang, Guang Li, Jia Shen, Jiayin Du, Xiaochen Lu, Xingliang Xiong

**Affiliations:** ^1^ Department of Medical Informatics, Chongqing Medical University, Chongqing, 400016, China

**Keywords:** cancer risk, toll-like receptor, TLR4, SNP, meta-analysis

## Abstract

Previous studies have explored the association between toll-like receptor 4 (TLR4) polymorphisms and risk of various cancers, but the results remained controversial. To obtain an assessment of the effect of TLR4 polymorphisms (rs4986790, rs4986791 and rs11536889) on cancer risk, fifty-five articles (containing 20107 cases and 28244 controls) were recruited for meta-analysis. Our result indicated that two Single Nucleotide Polymorphisms (SNP) in TLR4 were associated with decreased cancer risk for rs4986791: OR = 0.764, 95% CI: 0.652-0.894, *P =* 0.001 in allele model; OR = 0.769, 95%CI: 0.650-0.909, *P =* 0.002 in recessive model; OR = 0.505, 95% CI: 0.352-0.726, *P =* 0.000 in dominant model; for 11536889: OR = 0.927, 95% CI: 0.872–0.984, *P =* 0.013 in allele model; OR = 0.926, 95% CI: 0.862–0.944,*P =* 0.034 in recessive model. In terms of subgroup analyses sorted by ethnicity, only polymorphism of rs4986791 had a significant influence on decrease of cancer risk among both Caucasian and Asian populations. The findings suggested that TLR4 polymorphisms may serve as a genetic risk factor for cancers.

## INTRODUCTION

Toll-like receptors (TLRs) were important innate immune molecules which were used for specific recognition to adjust the adaptive immune response by identifying the pathogen-associated molecular patterns (PAMPs). In addition, TLRs stimulated the immune system and promoted the development of cancer due to its potential chronic inflammation [1–3]. In recent years, many research findings revealed the functions and molecular mechanisms of TLRs in cancers [4, 5], which indicated that TLRs may play a role in the cancer occurrence and development. There were 13 kinds of TLRs found in the mammal body, among them, only TLR2 and TLR4 can bond with glycosyl ligand and their intracellular adapters were mainly consist of Toll/IL-1 homologous receptor(TIR), myeloid differentiation factor 88 (My88), TIR domain containing adapter-inducing interferon-β (TRIF), TRIF-related adaptor molecule (TRAM) and MyD88 adaptor-like (MAL).

Recent studies indicated that TLR4 expressed on many cells as the bridge between the natural immunity and acquired immunity was able to produce proinflammatory factors and chemotactic factors after combining with ligands such as lipopolysaccharide(LPS) and teichoic acid [6]. Moreover, TLR4 signals were transmitted through MyD88-dependency and MyD88-independency. After combining with ligands, TLR4 transmitted the stimulation signals into cell nucleus which was activated by a series of protein cascade reactions [7]. Such behavior led to the activation of important immune gene transcription factors such as NF-κB, activator protein-1 and interferon regulatory factor (IRF) that induced the synthesis and release of relevant cell factors like IL-l, IL-2,TNF-α and IFN and enabled the acquired immune action by promoting the maturity of dendritic cells (DC), thus, stimulating a series of immune reactions with different pathogenic microorganism in the end [8]. The MyD88 dependent way mainly mediated the combination of interleukin-1 receptor-associated kinase-1 (IRAK-1), IRAK4 and TLR4, and dissociated IRAK-1 from receptor complex after its phosphorylation activating necrosis tumor factor-associated factor 6(TRAF-6) through binding with it. TRAF-6 then stimulated the inhibitor of nuclear factor kappa-B kinase (IKK) complex by exciting transforming growth factor-β-activating kinase 1 (TAK1), induced IκB phosphorylation to release and transmit NF-κB into cell nucleus enabling the relevant genetic transcription [9, 10]. The MyD88 independent way was mainly related to LPS-induced interferon induced protein-10 (IP-10), IFN-regulate gene 1 (IRG-l) and glucocorticoid attenuated responsegene-16 (GARG16) expressions and the DC maturity.

Numerous publications demonstrated that the existence of single nucleotide polymorphism (SNPs) made it possible for affecting TLR4 signaling, which was responsible for hyporesponsiveness and infection of fungus, virus and bacteria and the expression of TLR4 had a close association with the development of breast cancer [11–14], lung cancer [15], prostate cancer [16–21], colorectal cancer [22–26] and liver cancer [25, 27–29]. Due to the inconsistence of these findings, arising probably from inadequacy of sample size, we made a comprehensive study to better understand the association between TLR4 gene and cancer risk. There were 20107 cases and 28244 controls totally in the research and for rs2986790 and rs2986791, the amount of cases and controls were 9105 and 11338, 4416 and 7379, respectively. There were 6586 cases and 9527 controls to evaluate the association between rs11536889 polymorphism and the risk of cancer.

## RESULTS

### Studies included in the meta-analysis

**Figure 1:**
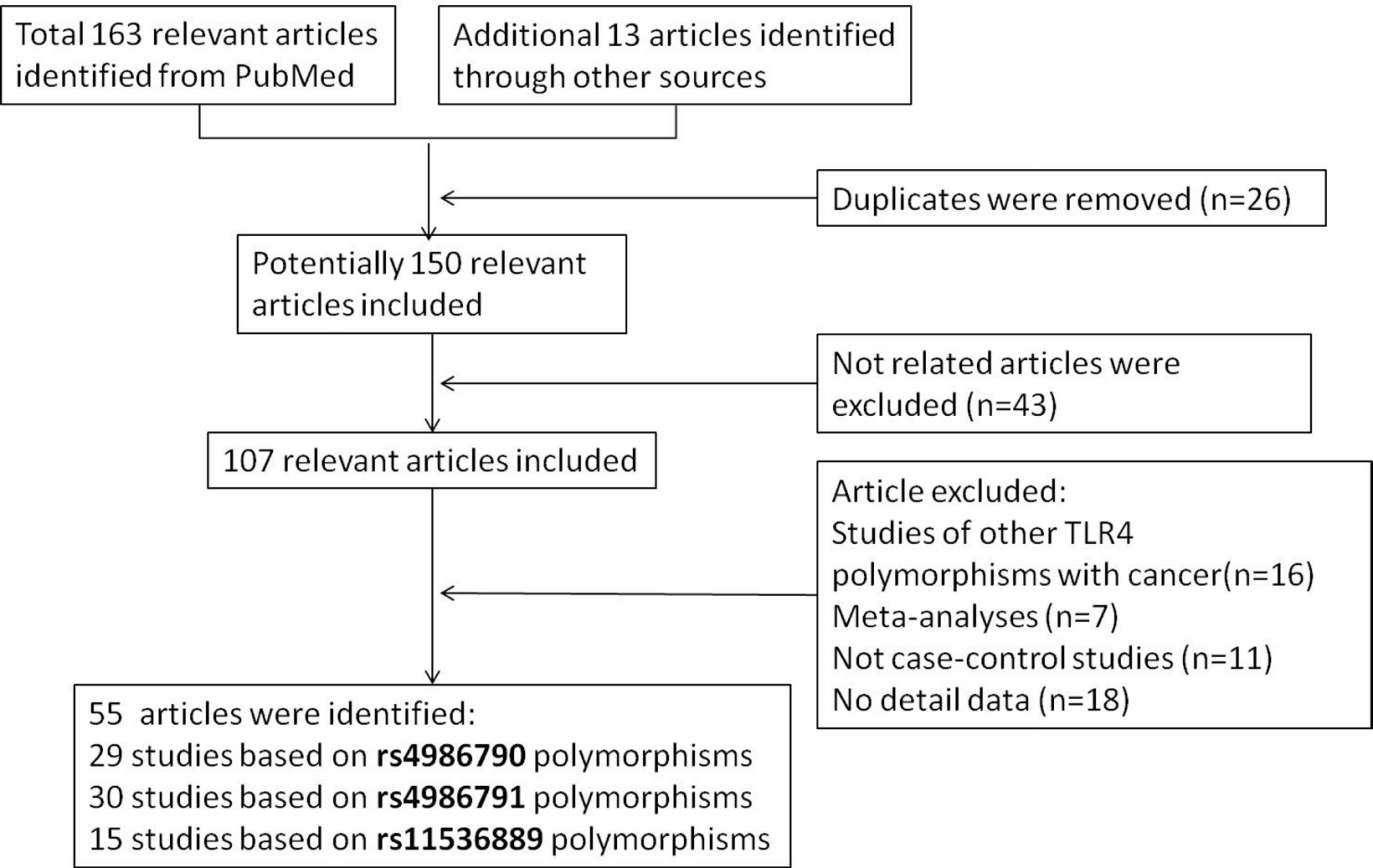
Flow diagram of included and excluded publications

In the beginning, there were 166 relevant articles searched mainly from PubMed, of which 26 duplicates were excluded. And 43 articles were eliminated because of their irrelevance with cancer. After getting rid of studies focusing on other gene instead of TLR4, meta-analysis, studies lacking comparison between case and control and studies having few valuable data, 55 publications were eventually included. As elaborated in Figure 1, the flow chart of selecting, 29, 30, 15 articles were suitable for investigating the relation between rs2986790, rs2986791 and rs11536889 polymorphisms and cancer risk, respectively. In addition, detail information on included articles were shown in Supplementary Table 1, while studies disagreeing with HWE containing seven for rs4986790, three for rs4986791 and one for rs11536889 were shown in bold.

### Meta-analysis results

TLR4 rs4986790 polymorphism and cancer risk. Before the analysis, we tested the sensitivity of this SNP and found an article affecting the sensitivity greatly through leave-one-out method. Therefore, this article written by Hold GL1 [30] about gastric cancer was excluded in our study. We then evaluated the association between rs4986790 polymorphism and the risk of cancer through analyzing data gained from 20 articles, containing 9105 cases and 11338 controls. However, few connection between rs4986790 polymorphism and risk of cancer was found under three genetic models shown in Table 1 and Supplementary Figure 1.

**Table 1:** Meta-analysis of the association between rs4986790 polymorphism and cancer risk

Population	*N*	A vs. G(Allele model)			AA vs. AG + GG (Recessive model)			AA + AG vs. GG (Dominant model)		
		OR(95%CI)	POR	Ph	OR (95%CI)	POR	Ph	OR (95%CI)	POR	Ph
Overall	21	0.871 (0.763–0.995)	0.289	0.126	0.919 (0.839–1.005)	0.065	0.212	1.320 (0.855–2.039)	0.293	0.804
Caucasian	14	0.946 (0.854–1.048)	0.289	0.048	0.925 (0.831–1.030)	0.155	0.087	1.497 (0.853–2.626)	0.254	0.584
Asian	3	0.771 (0.571–1.042)	0.091	0.886	0.780 (0.526–2.363)	0.134	0.785	0.469 (0.134–1.642)	0.236	0.835
African	1	1.227 (0.602–2.497)	0.573	–	1.115 (0.526–2.363)	0.776	–	3.628 (0.172–76.396)	0.407	–
Mixed	3	0.965 (0.795–1.170)	0.716	0.282	0.941 (0.768–1.152)	0.551	0.246	1.499 (0.593–3.789)	0.392	0.966

The results were in bold if the P_OR_< 0.05. Odd ratio (OR), 95% confidence interval (95% CI) and P_OR_ were tested to evaluate the association, while P_h_ was examined for heterogeneity

TLR4 rs4986791 polymorphism and cancer risk. To investigate the relation between the polymorphism and cancer risk, we searched 27 studies for useful data and 4416 cases and 7379 controls were analyzed. Table 2 and Supplementary Figure 2 demonstrated rs4986791 polymorphism always had a association with decrease of cancer risk among overall population(allele model: OR = 0.764, 95% CI: 0.652–0.894, *P* = 0.001; recessive model: OR = 0.769, 95% CI: 0.650–0.909, *P* = 0.002; dominant model: OR = 0.505, 95% CI: 0.352–0.726, *P* = 0.000), Caucasian population (allele model: OR = 0.790, 95% CI: 0.646–0.966, *P* = 0.022; recessive model: OR = 0.798, 95% CI: 0.649–0.982, *P* = 0.033; dominant model: OR = 0.430, 95% CI: 0.205–0.904, *P* = 0.026) and Asian population (allele model: OR = 0.633, 95% CI: 0.555–0.793, P = .000; recessive model: OR = 0.656, 95% CI: 0.548–0.786, *P* = 0.000; dominant model: OR = 0.532, 95% CI: 0.351–0.806, *P* = 0.003) in three genetic models.

**Table 2:** Meta-analysis of the association between rs4986791 polymorphism and cancer risk

Population	*N*	C vs. T(Allele model)			CC vs. CT + TT (Recessive model)			CC + CT vs. TT (Dominant model)		
		OR(95%CI)	P_OR_	P_h_	OR (95% CI)	P_OR_	P_h_	OR(95%CI)	P_OR_	Ph
Overall	27	0.764 (0.652–0.894)	0.001	0.031	0.769 (0.650–0.909)	0.002	0.035	0.505 (0.352–0.726)	0.000	0.777
Caucasian	19	0.790 (0.646–0.966)	0.022	0.082	0.798 (0.649–0.982)	0.033	0.095	0.430 (0.205–0.904)	0.026	0.635
Asian	5	0.633 (0.555–0.793)	0.000	0.336	0.656 (0.548–0.786)	0.000	0.397	0.532 (0.351–0.806)	0.003	0.663
African	1	2.090 (0.809–5.399)	0.128	–	2.165 (0.822–5.704)	0.118	–	–	–	–
Mixed	2	1.397 (0.224–8.700)	0.720	0.117	1.408 (0.219–9.065)	0.719	0.114	–	–	–

The results were in bold if the P_OR_< 0.05 Odd ratio (OR), 95% confidence interval (95% CI) and P_OR_ were tested to evaluate the association, while P_h_ was examined for heterogeneity.

TLR4 rs11536889 polymorphism and cancer risk. Taking 14 articles, containing 6586 cases and 9527 controls, into consideration, we noticed a connection between rs11536889 polymorphism and decreased risk of cancer in overall population under two genetic models (allele model: OR = 0.927, 95% CI: 0.872–0.984, *P* = 0.013; recessive model: OR = 0.926, 95% CI: 0.862–0.944, *P* = 0.034). The similar association was also found for rs11536889 in Asian population under allele model (OR = 0.916, 95% CI = 0.845–0.992, *P* = 0.031) and dominant model (OR = 0.777, 95% CI = 0.640–0.943, *P* = 0.011) illustrated in Table 3 and Supplementary Figure 3.

**Table 3:** Meta-analysis of the association between rs11536889 polymorphism and cancer risk

Population	*N*	G vs. C(Allele model)			GG vs. GC+CC (Recessive model)			GG+GC vs. CC(Dominant model)		
		OR(95%CI)	POR	Ph	OR(95%CI)	POR	Ph	OR(95%CI)	POR	Ph
Overall	14	0.927 (0.872–0.984)	0.013	0.400	0.926 (0.862–0.944)	0.034	0.446	0.853 (0.723–1.007)	0.060	0.508
Caucasian	6	0.941 (0.858–1.031)	0.193	0.722	0.919 (0.829–1.018)	0.106	0.529	1.082 (0.790–1.483)	0.623	0.824
Asian	8	0.916 (0.845–0.992)	0.031	0.159	0.932 (0.844–1.029)	0.164	0.264	0.777 (0.640–0.943)	0.011	0.438

The results were in bold if the *P*_OR_< 0.05. Odd ratio (OR), 95% confidence interval (95% CI) and P_OR_ were tested to evaluate the association, while P_h_ was examined for heterogeneity.

**Table 4:** Association between TLR4 polymorphisms and overall cancer risk by cancer type

Polymorphisms	Ethnicity	Cancer Type	*N*	Allele model			Recessive model			Dominant model		
				OR (95%CI)	POR	I^2^	OR (95%CI)	POR	I^2^	OR(95%CI)	POR	I^2^
**rs4986790**	Caucasian	Male- specific	3	1.221 (0.921–1.619)	0.166	25.4%	1.162 (0.864–1.563)	0.320	25.8%	5.983 (1.302–27.488)	0.021	0.0%
		Digestive	5	0.898 (0.691–1.166)	0.420	21.7%	0.909 (0.729–1.134)	0.400	3.9%	0.791 (0.204–3.064)	0.734	0.0%
		Blood	2	0.965 (0.795–1.172)	0.722	0.0%	0.957 (0.782–1.172)	0.673	0.0%	1.207 (0.349–4.178)	0.766	0.0%
		Female- specific	2	**0.639** **(0.478–0.854)**	**0.002**	**0.0%**	**0.611** **(0.447–0.837)**	**0.002**	**0.0%**	0.679 (0.221–2.090)	0.500	0.0%
		Others	1	0.887 (0.650–1.210)	0.448	–	0.843 (0.610–1.164)	0.299	–	7.229 (0.373–140.201)	0.191	–
		Overall	13	0.924 (0.796–1.072)	0.297	43.2%	0.906 (0.782–1.049)	0.186	37.0%	1.310 (0.698–2.456)	0.400	0.0%
**rs4986791**	Asian	Others	2	**0.592** **(0.422–0.829)**	**0.002**	**0.0%**	**0.581(0.406–0.833)**	**0.003**	**0.0%**	0.361 (0.069–1.883)	0.227	0.0%
		Digestive	1	0.549 (0.385–0.783)	0.001	–	0.534 (0.360–0.792)	0.002	–	0.244 (0.051–1.162)	0.076	–
	Caucasian	Digestive	13	**0.720** **(0.574–0.902)**	**0.004**	**33.9%**	**0.721** **(0.577–0.901)**	**0.004**	**27.5%**	0.479 (0.159–1.448)	0.192	3.7%
		Female- specific	3	0.851 (0.559–1.296)	0.453	0.0%	0.887 (0.569–1.381)	0.594	0.0%	0.327 (0.047–2.246)	0.255	0.0%
		Others	3	1.692 (0.781–3.666)	0.182	25.7%	1.849 (0.923–3.703)	0.083	5.2%	0.159 (0.006–3.967)	0.263	–
		Overall	19	**0.790** **(0.646–0.966)**	**0.002**	**32.9%**	**0.798** **(0.649–0.982)**	**0.033**	**31.3%**	**0.401** **(0.162–0.993)**	**0.048**	**0.0%**
**rs11536889**	Asian	Digestive	7	0.937 (0.844–1.041)	0.226	21.4%	0.960 (0.852–1.082)	0.503	11.6%	0.821 (0.658–1.026)	0.083	0.0%
		Others	1	0.791 (0.657-0.951)	0.013	–	0.797 (0.631–1.008)	0.059	–	**0.627** **(0.415–0.947)**	0.026	–
		Overall	8	0.907 (0.818–1.006)	0.066	33.7%	0.928 (0.826–1.041)	0.202	20.9%	**0.773** **(0.636–0.940)**	0.010	0.0%
	Caucasian	Male- specific	4	0.946 (0.854–1.048)	0.287	0.0%	0.927 (0.809–1.062)	0.273	25.0%	1.071 (0.749–1.533)	0.706	0.0%
		Digestive	2	0.924 (0.751–1.136)	0.454	0.0%	0.891 (0.706–1.126)	0.334	0.0%	1.124 (0.571-2.212)	0.735	0.0%
		Overall	6	0.941 (0.859-1.032)	0.198	0.0%	0.920 (0.830–1.019)	0.110	0.0%	1.083 (0.789-1.486)	0.623	0.0%

The results were in bold if the *P*_OR_< 0.05. Odd ration (OR), 95% confidence interval (95%CI) and POR were tested to evaluate the association, while I^2^ was examined for heterogeneity.

### Evaluation of heterogeneity

As shown in Tables 1–3 and Supplementary Table 2, slight heterogeneities existed in overall comparisons (P_h_ = 0.126, *I*^2^ = 27.3% for rs4986790; P_h_ = 0.031, *I*^2^ = 36.6% for rs4986791; P_h_ = 0.400, *I*^2^ = 4.7% for rs11536889). We performed logistical meta-regression to evaluate the source of heterogeneity among all studies and found that ethnicity had a significant influence on heterogeneity, while cancer type, number of alleles, sample size and genotyping methods could not greatly influence the initial heterogeneity. According to Cochrane handbook [31], however, the heterogeneity whose value of *I*^2^ is lower than 50% is acceptable confirming the credibility of our study.

### Publication bias and sensitivity analysis

**Figure 2:**
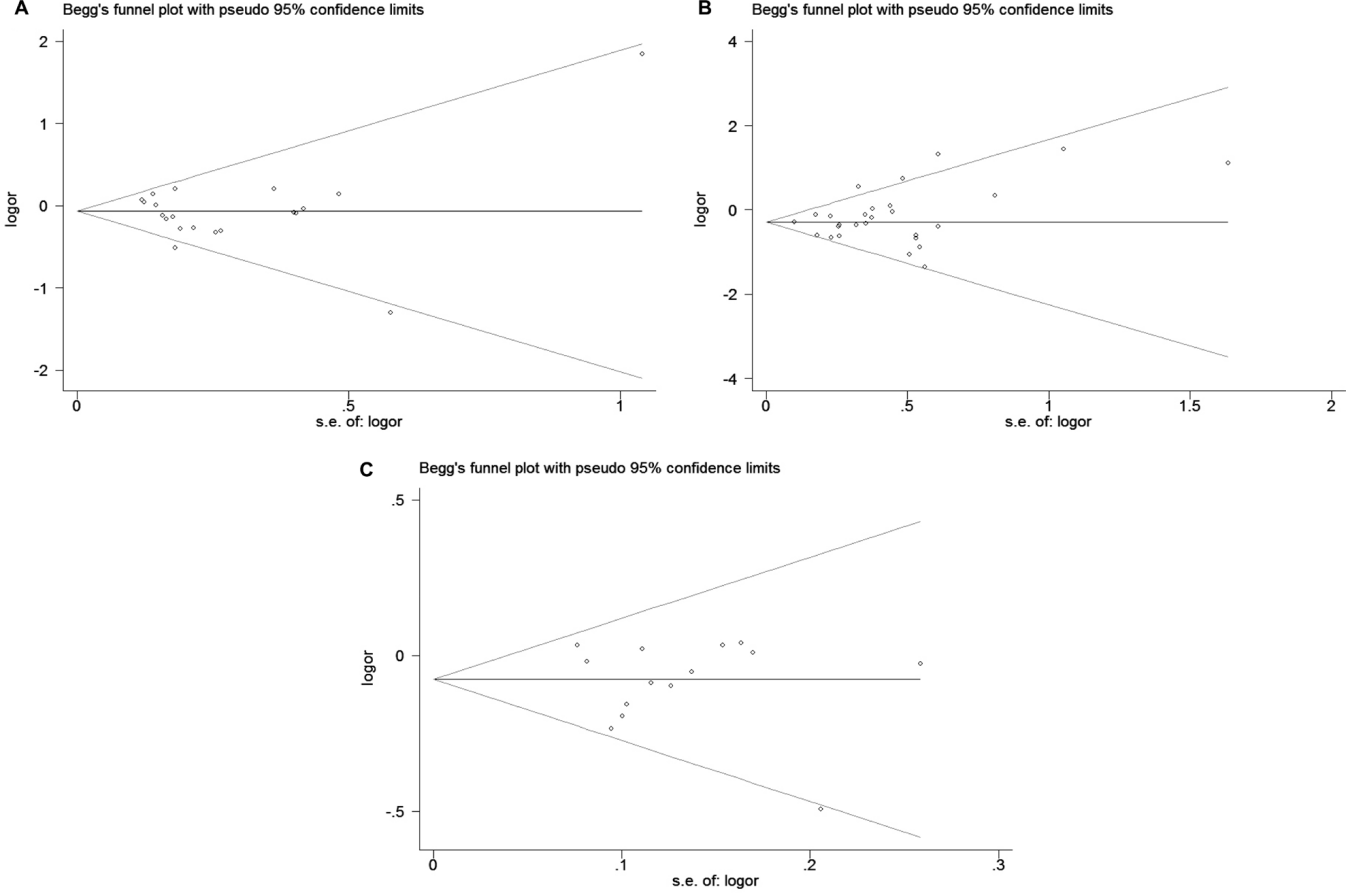
Begg’s funnel plot for publication bias test of (**A**) rs4986790, (**B**) rs4986791 and (**C**) rs11536889 polymorphisms. Each point stand for an individual article in overall population under allele model. s.e., standardized effect.

**Figure 3:**
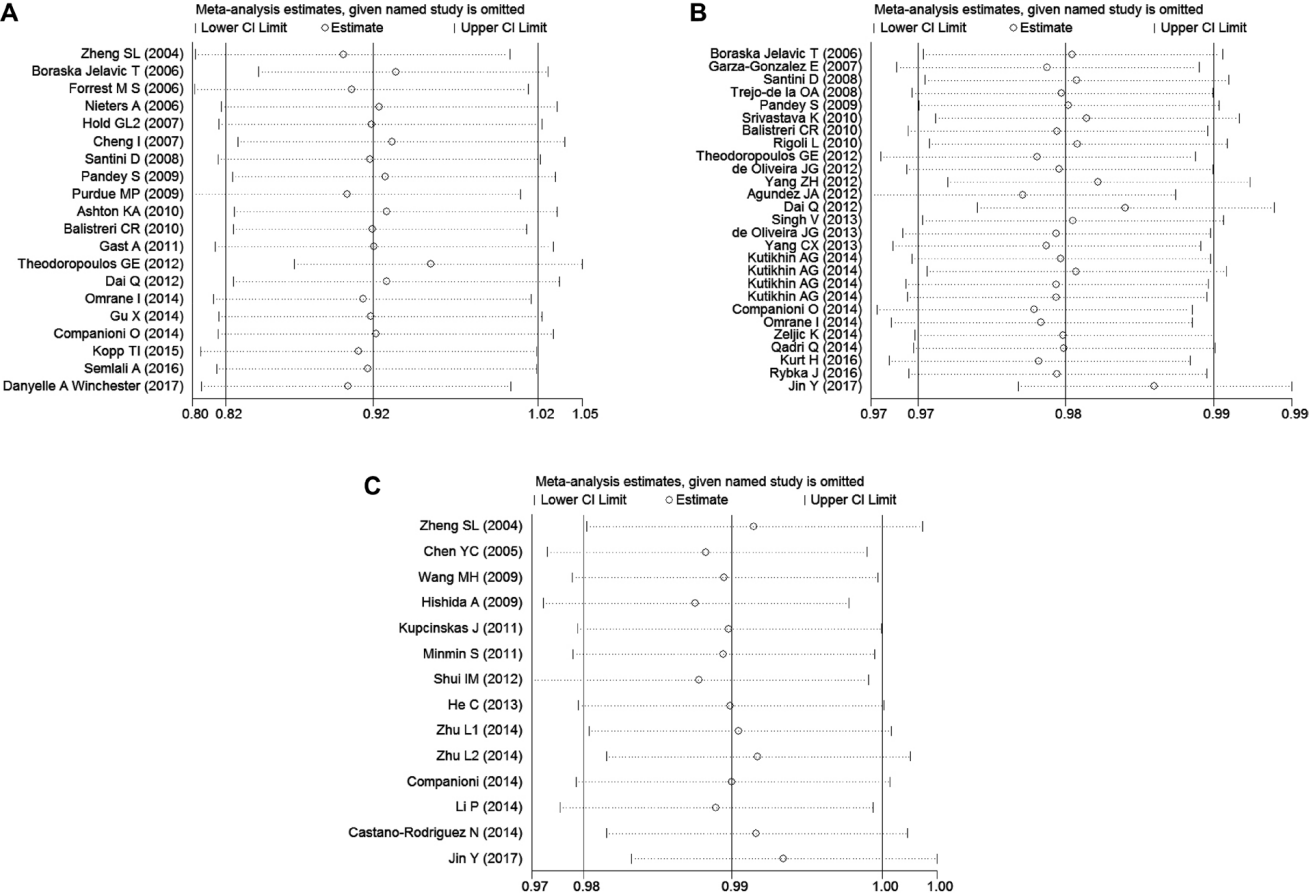
Sensitivity analysis to assess the stability of the meta-analysis about (**A**) rs4986790, (**B**) rs4986791 and (**C**) rs11536889 polymorphisms in overall population under allele model.

The publication bias of included literatures was evaluated through the Begg’s test under allele model. As displayed in Figure 2, there was no evident publication bias in these studies (*P* = 0.496 for rs4986790, after the article of Hold GL was excluded from this study; *P* = 0.297 for rs4986791; *P* = 0.827 for rs11536889, respectively). The leave-one-out sensitivity analysis was also performed to examine the effects of individual article on the pooled ORs. The result, illustrated in Figure 3, indicated that the pooled OR had no evident change after removing any studies.

## DISCUSSION

In this study, we conducted a meta-analysis of 55 independent articles concerning the association between TLR4 polymorphisms and cancer risk. As a result, rs4986791 polymorphism had a significant association with decreased cancer risk under 3 genetic model. And similar association was also found between rs11536889 and risk of cancer in overall and Asian populations.

TLR4 gene, located in chromosome 9q32-q33, was an important receptor of lipopolysaccharide and played a key role in immune responses thorough activation of nuclear factor kappa-light-chain-enhancer [32, 33]. It, thus, became a focus on infection and immunity in current studies. Polymorphisms of rs4986790 and rs4986791 in exon 3, leading respectively to the substitution of Asp299Gly and Thr399Ile amino acid and downregulating the expression of several genes in the TLR4 TRAM/TRIF signaling pathway [34], was associated with endotoxin hyporesponsiveness [35], gram-negative septic shock [36] and atherogenesis [37]. However, analyses focusing on them did not reach a consensus on cancer risk and some proved they had a nonnegligible impact on breast cancer [11, 13] and digestive cancer [23, 26], while the others believed that no evident could support significant association with gastric cancer [38, 39], bladder cancer [40], prostate cancer [20], leukemia [41]. To minimized the effects of sample size, a large amount of publications on variant cancers were collected in this study and the consequence reflected there was a significant association between rs4986791 polymorphism and the decrease of cancer risk while polymorphism of rs4986790 was found irrelevant to cancer risk. The influence of rs11536889, whose genetic variation contributed to translational regulation of TLR4, possibly by binding to microRNAs [42], on decrease of cancer risk was also prominent which was consistent with results of previous articles [43, 44].

As for stratified analyses by ethnicity, polymorphism of rs4986791 had a prominent effect on decreased cancer risk of Caucasian and Asian. Also, decreased cancer risk among Asian population was associated with rs11536889 polymorphism.

In terms of subgroup analyses classified by race and cancer type, we found that Caucasian female-specific cancer had a conspicuous association with polymorphisms of rs4986790 and rs4986791 and Asian digestive cancer was significantly influenced by rs4986791 polymorphism. There was no evidence for another association between these SNPs and cancer risk.

In summary, this meta-analysis illustrated polymorphisms of TLR4 rs4986791 and rs11536889 might make contributions to slow the formation and development of cancer, whereas rs4986790 was not strongly associated with cancer risk. Due to the limit of sample size of African population in this study, there is still a requirement of researches including more data for getting insight into association between TLR4 polymorphisms and cancer risk.

## MATERIALS AND METHODS

### Identification of eligible studies

To investigate the association between TLR4 polymorphisms and cancer risk, we searched PubMed and some other databases for relevant articles, ranging from 2000 to 2017 (the last article was published on June 1^st^, 2017 ). The key words used in searching were ‘TLR4 or toll-like receptor’ and ‘cancer’ and ‘SNP or polymorphism’. As illustrated in Figure 1, 163 articles from PubMed and 13 articles from other sources were included at first and 55 articles were included eventually.

### Data extraction

Articles meeting all the requirements below were eligible for the analysis:(a) the studies focused on TLR4 and cancer risk;(b) each publication was case-control study and had valuable data for calculating genotype counts;(c) the cancers were classified by the experienced pathologist, according to the World Health Organization (WHO) criteria; (d) the articles were written in English or Chinese. The following information is shown in our study: first author, journal, year of publication, ethnicity, cancer type, sample size, genotype and allele frequency.

### Statistical analysis

In this study, we used the software Stata 12.0 (Stata corporation, College Station, TX, USA) for meta-analysis of the association between TLR4 polymorphisms and cancer risk, which was estimated by odds ratio (OR) and 95% confidence intervals (CI). In addition, we conducted stratification analysis by ethnicity (Caucasian, Asian, African and Mixed populations) and cancer type (digestive cancer was consist of hepatocellular cancer, oral cancer, gastric cancer, esophageal cancer, colon cancer and rectal cancer; male-specific cancer was consist of prostate cancer; female-specific cancer was consist of breast cancer, cervical cancer, endometrial cancer and ovarian cancer; blood cancer was consist of lymphoma and leukemia; one cancer type was ranked into others if the number of studies was less than 3 ) to understand the effects of them on the association. Hardy-Weinberg equilibrium (HWE) was estimated for each study by Pearson Chi-square test in control group and *P* < 0.05 was regard a significant departure from HWE. Allele model, recessive model and dominant model were compared to evaluate the association between TLR4 polymorphisms (rs4986790, rs4986791 and rs11536889) and the risk of cancer.

We adopted Begg’s test and Funnel plots for evaluation of publication bias of this study and exclusion of one article at a time leading to new pooled ORs, which showed the influence of individual article on the overall consequence. Moreover, to examine heterogeneity among all the articles, the *Q*-test and *I*^2^ statistics were selected. When the heterogeneity was obvious (P_h_> 0.05), the mixed effects model (the Mantel-Haenszel method) was used for the summary OR value. Otherwise, the random effects model (the DerSimonian and Laird method) took the place of it.

## SUPPLEMENTARY MATERIALS FIGURES AND TABLES




