# Supplementary material for: Comprehensive assessment of association between TLR4 gene polymorphisms and cancer risk: a systematic meta-analysis
Source: Oncotarget. 2017 Oct 6;8(59):100593–602. doi: 10.18632/oncotarget.21543 (PMC5725046; doi:10.18632/oncotarget.21543)
Supplement: Supplementary file 1 [file oncotarget-08-100593-s001.pdf]

# Genetic predisposition toward suicidal ideation in patients with acute coronary syndrome

## SUPPLEMENTARY MATERIALS

**Supplementary Table 1: The basic information of studies included in the meta-analysis.** See Supplementary_Table_ 1

**Supplementary Table 2: Heterogeneity of polymorphisms of TLR4 gene.**

| Population | *N* | Allele model | | Recessive model | | Dominant model | |
|---|---|---|---|---|---|---|---|
| | | $P_h$ | $I^2$ | $P_h$ | $I^2$ | $P_h$ | $I^2$ |
| **rs4986790** | | | | | | | |
| Overall | 20 | 0.126 | 27.3% | 0.212 | 19.5% | 0.800 | 0.0% |
| Caucasian | 13 | 0.048 | 43.2% | 0.087 | 37.0% | 0.582 | 0.0% |
| Asian | 3 | 0.886 | 0.0% | 0.785 | 0.0% | 0.835 | 0.0% |
| African | 1 | — | — | — | — | — | — |
| Mixed | 3 | 0.282 | 20.9% | 0.246 | 28.7% | 0.966 | 0.0% |
| **rs4986791** | | | | | | | |
| Overall | 27 | 0.031 | 36.6% | 0.035 | 35.9% | 0.777 | 0.0% |
| Caucasian | 19 | 0.082 | 32.9% | 0.095 | 31.3% | 0.635 | 0.0% |
| Asian | 5 | 0.336 | 12.3% | 0.397 | 1.6% | 0.663 | 0.0% |
| African | 1 | — | — | — | — | — | — |
| Mixed | 2 | **0.117** | **59.2%** | **0.114** | **60.0%** | — | — |
| **rs11536889** | | | | | | | |
| Overall | 14 | 0.400 | 4.7% | 0.446 | 0.0% | 0.508 | 0.0% |
| Caucasian | 6 | 0.722 | 0.0% | 0.529 | 20.9% | 0.824 | 0.0% |
| Asian | 8 | 0.159 | 33.7% | 0.264 | 0.2% | 0.438 | 0.0% |

The results were in bold if $I^2 > 50\%$, which was not acceptable.

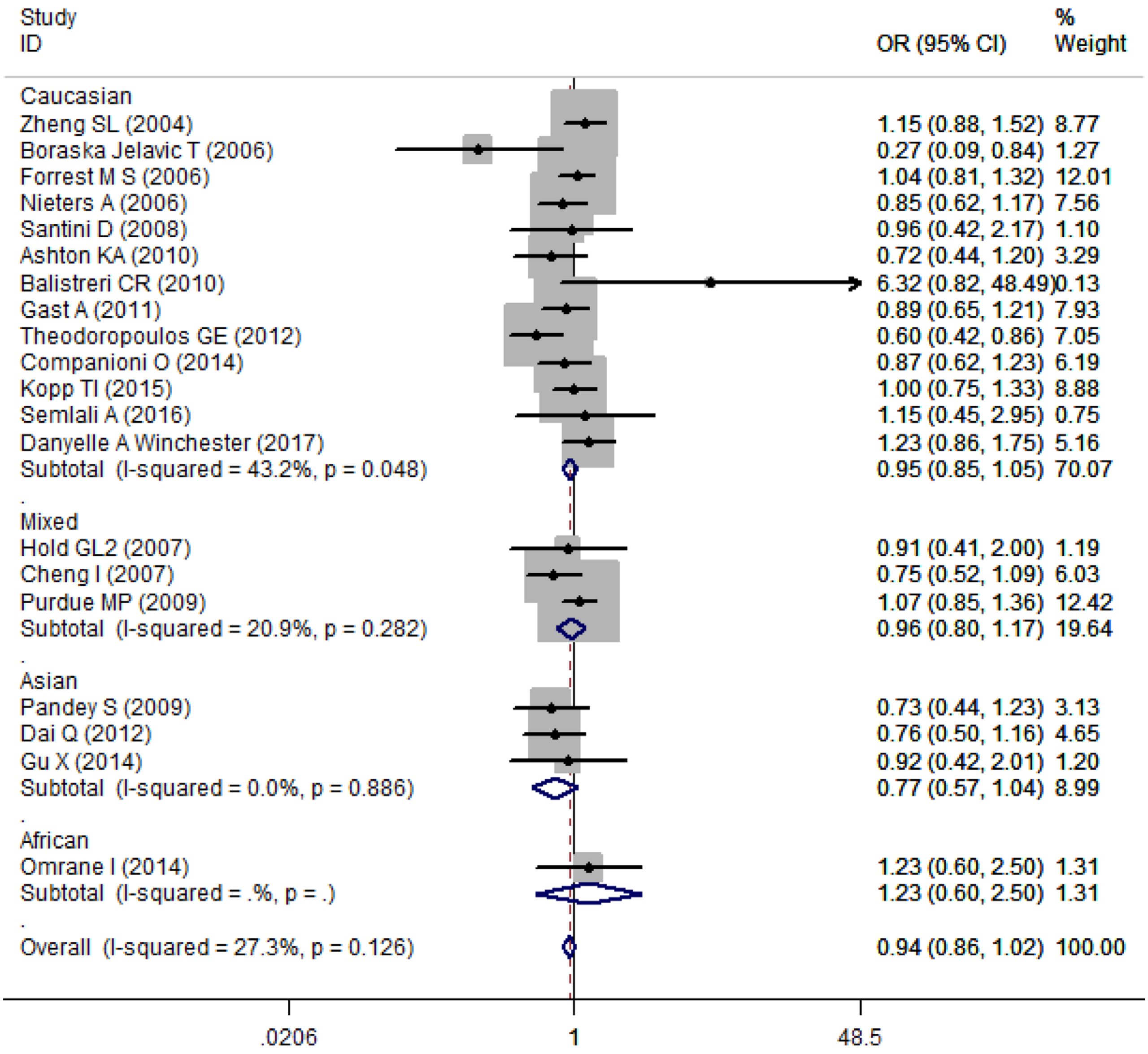


**Supplementary Figure1: Forest plot for meta-analysis of the association between rs4986790 polymorphism and cancer risk.**

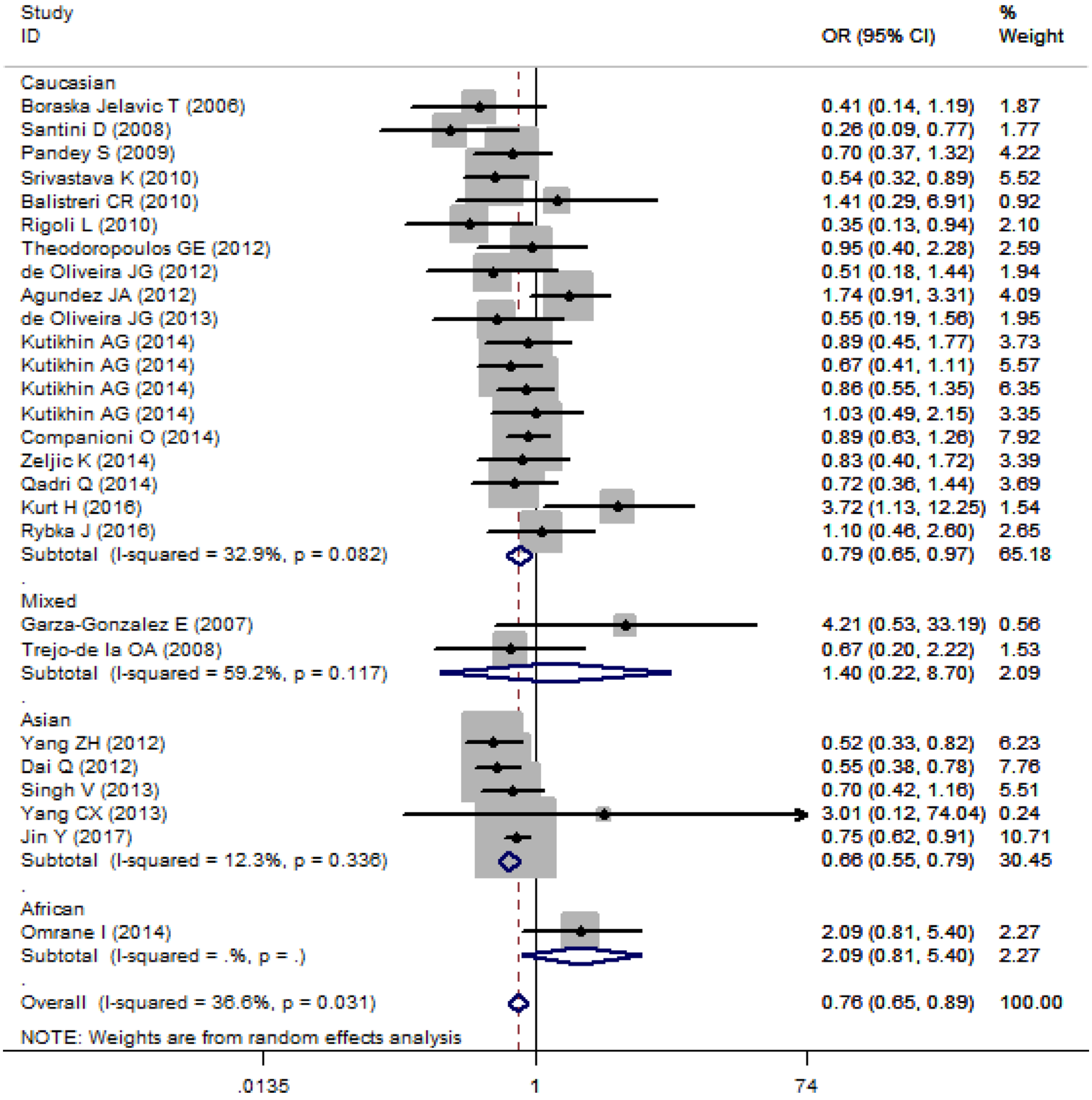


**Supplementary Figure 2: Forest plot for meta-analysis of the association between rs4986791 polymorphism and cancer risk.**

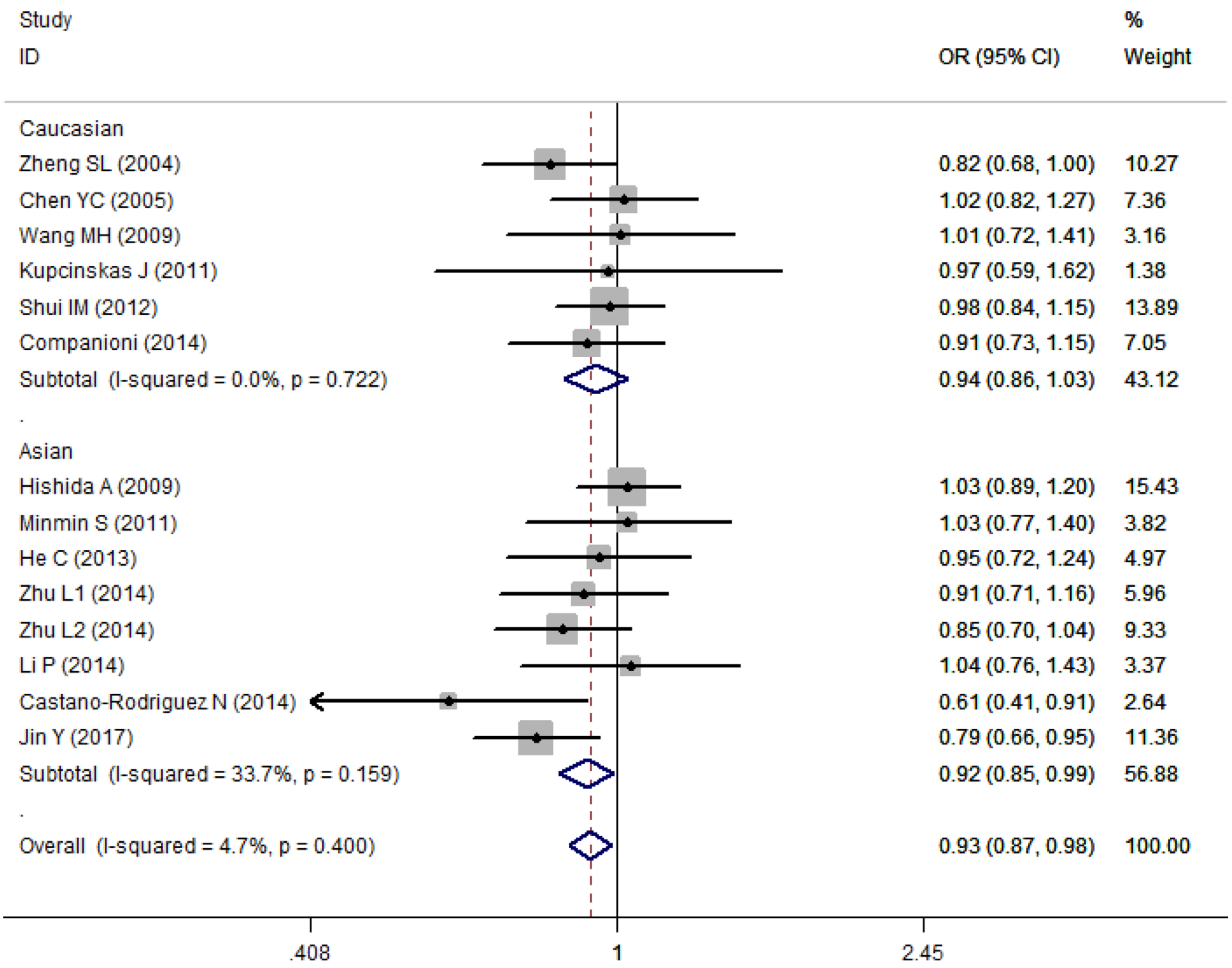


**Supplementary Figure 3: Forest plot for meta-analysis of the association between rs11536889 polymorphism and cancer risk.**
