# Supplementary material for: Comprehensive assessment of association between TLR4 gene polymorphisms and cancer risk: a systematic meta-analysis
Source: Oncotarget. 2017 Oct 6;8(59):100593–602. doi: 10.18632/oncotarget.21543 (PMC5725046; doi:10.18632/oncotarget.21543)
Supplement: Supplementary file 2 [file oncotarget-08-100593-s002.docx]

**Supplementary Table 1: The basic information of studies included in the meta-analysis. The data which had a significant departure from HWE were shown in bold.**

| **Polymorphisms and study** | | **Journal** | | **Year** | | **Ethnicity** | | **Cancer type** | | **Sample size** | | | | **Genotypes** | | | | | | | | | | | | **Allele frequencies(%)** | | | | | | | |
| --- | --- | --- | --- | --- | --- | --- | --- | --- | --- | --- | --- | --- | --- | --- | --- | --- | --- | --- | --- | --- | --- | --- | --- | --- | --- | --- | --- | --- | --- | --- | --- | --- | --- |
|  |  |  |  |  |  |  |  |  |  | **Cases** | | **Controls** | | **Cases** | | | | | | **Controls** | | | | | | **Cases** | | | | **Controls** | | | |
| **rs4986790** | |  | |  | |  | |  | |  | |  | | **AA** | | **AG** | | **GG** | | **AA** | | **AG** | | **GG** | | **A** | | **G** | | **A** | | **G** | |
| Zheng SL[16] | | Cancer Res | | 2004 | | Caucasian | | Prostate cancer | | 1378 | | 777 | | 1241 | | 136 | | 1 | | 693 | | 79 | | 5 | | 0.950 | | 0.050 | | 0.943 | | 0.057 | |
| **Chen YC[45]** | | **Cancer Res** | | **2005** | | **Caucasian** | | **Prostate cancer** | | **657** | | **669** | | **588** | | **66** | | **3** | | **605** | | **59** | | **5** | | **0.945** | | **0.055** | | **0.948** | | **0.052** | |
| Boraska Jelavic T[46] | | Clin Genet | | 2006 | | Caucasian | | Colorectal cancer | | 89 | | 88 | | 77 | | 10 | | 2 | | 84 | | 4 | | 0 | | 0.921 | | 0.079 | | 0.977 | | 0.023 | |
| Forrest M S[47] | | Br J Haematol | | 2006 | | Caucasian | | Lymphoma | | 903 | | 1432 | | 794 | | 106 | | 3 | | 1254 | | 172 | | 6 | | 0.938 | | 0.062 | | 0.936 | | 0.064 | |
| Nieters A[48] | | Genes Immun | | 2006 | | Caucasian | | Lymphoma | | 675 | | 668 | | 590 | | 84 | | 1 | | 596 | | 71 | | 1 | | 0.936 | | 0.064 | | 0.945 | | 0.055 | |
| Hold GL1[34] | | Gastroenterology | | 2007 | | Caucasian | | Gastric cancer | | 496 | | 630 | | 414 | | 79 | | 3 | | 581 | | 47 | | 2 | | 0.914 | | 0.086 | | 0.960 | | 0.040 | |
| Hold GL2[34] | | Gastroenterology | | 2007 | | Mixed | | Oesophageal cancer | | 107 | | 211 | | 97 | | 10 | | 0 | | 194 | | 16 | | 1 | | 0.953 | | 0.047 | | 0.957 | | 0.043 | |
| Cheng I[21] | | Cancer Epidemiol  Biomarkers Prev | | 2007 | | Mixed | | Prostate cancer | | 506 | | 506 | | 439 | | 66 | | 1 | | 456 | | 48 | | 2 | | 0.933 | | 0.067 | | 0.949 | | 0.051 | |
| Santini D[49] | | Clin Exp Immunol | | 2008 | | Caucasian | | Gastric cancer | | 171 | | 151 | | 159 | | 11 | | 1 | | 140 | | 11 | | 0 | | 0.962 | | 0.038 | | 0.964 | | 0.036 | |
| Pandey S[50] | | Gynecol Oncol | | 2009 | | Asian | | Cervical cancer | | 150 | | 150 | | 114 | | 35 | | 1 | | 123 | | 26 | | 1 | | 0.877 | | 0.123 | | 0.907 | | 0.093 | |
| Purdue MP[51] | | Carcinogenesis | | 2009 | | Mixed | | Non-Hodgkin lymphoma | | 1334 | | 1265 | | 1195 | | 133 | | 6 | | 1126 | | 131 | | 8 | | 0.946 | | 0.054 | | 0.942 | | 0.058 | |
| Ashton KA[52] | | BMC Cancer | | 2010 | | Caucasian | | Endometrial cancer | | 191 | | 291 | | 163 | | 25 | | 3 | | 258 | | 31 | | 2 | | 0.919 | | 0.081 | | 0.940 | | 0.060 | |
| Balistreri CR[53] | | Curr Pharm Des | | 2010 | | Caucasian | | Prostate cancer | | 50 | | 125 | | 49 | | 1 | | 0 | | 111 | | 13 | | 1 | | 0.990 | | 0.010 | | 0.940 | | 0.060 | |
| Gast A[54] | | PLoS ONE | | 2011 | | Caucasian | | Malignant melanoma | | 756 | | 735 | | 665 | | 91 | | 0 | | 659 | | 73 | | 3 | | 0.940 | | 0.060 | | 0.946 | | 0.054 | |
| Theodoropoulos GE[12] | | Breast | | 2012 | | Caucasian | | Breast cancer | | 261 | | 480 | | 201 | | 57 | | 3 | | 412 | | 63 | | 5 | | 0.879 | | 0.121 | | 0.924 | | 0.076 | |
| **Yang ZH[55]** | | **Cancer Sci** | | **2012** | | **Asian** | | **Nasopharyngeal cancer** | | **236** | | **287** | | **205** | | **29** | | **2** | | **250** | | **33** | | **4** | | **0.930** | | **0.070** | | **0.929** | | **0.071** | |
| Dai Q[56] | | Chin J Pathophysiol | | 2012 | | Asian | | Colorectal cancer | | 268 | | 268 | | 219 | | 44 | | 5 | | 228 | | 38 | | 2 | | 0.899 | | 0.101 | | 0.922 | | 0.078 | |
| **Priyadarshini A[20]** | | **India J Urol** | | **2013** | | **Caucasian** | | **Prostate cancer** | | **198** | | **200** | | **157** | | **32** | | **9** | | **173** | | **20** | | **7** | | **0.874** | | **0.126** | | **0.915** | | **0.085** | |
| **Shen Y[40]** | | **Pathol Oncol Res** | | **2013** | | **Asian** | | **Bladder cancer** | | **436** | | **522** | | **431** | | **2** | | **3** | | **519** | | **1** | | **2** | | **0.991** | | **0.009** | | **0.995** | | **0.005** | |
| **Continued** | |  | |  | |  | |  | |  | |  | |  | |  | |  | |  | |  | |  | |  | |  | |  | |  | |

| **Polymorphisms and study** | **Journal** | **Year** | **Ethnicity** | **Cancer type** | **Sample size** | | **Genotypes** | | | | | | **Allele frequencies(%)** | | | |
| --- | --- | --- | --- | --- | --- | --- | --- | --- | --- | --- | --- | --- | --- | --- | --- | --- |
|  |  |  |  |  | **Cases** | **Controls** | **Cases** | | | **Controls** | | | **Cases** | | **Controls** | |
| **Pimentel-Nunes P[25]** | **Dig Liver Dis** | **2013** | **Caucasian** | **Colorectal cancer** | **184** | **191** | **169** | **0** | **15** | **186** | **0** | **5** | **0.918** | **0.082** | **0.974** | **0.026** |
| Omrane I[26] | Tumor Biol | 2014 | African | Colorectal cancer | 100 | 140 | 87 | 13 | 0 | 120 | 18 | 2 | 0.935 | 0.065 | 0.921 | 0.079 |
| Gu X[57] | Asian Pac J  Cancer Prev | 2014 | Asian | Non-Hodgkin lymphoma | 157 | 435 | 149 | 7 | 1 | 413 | 21 | 1 | 0.971 | 0.029 | 0.974 | 0.026 |
| Companioni O[58] | Int J Cancer | 2014 | Caucasian | Gastric cancer | 361 | 1270 | 316 | 45 | 0 | 1134 | 133 | 3 | 0.938 | 0.062 | 0.945 | 0.055 |
| Kopp TI[59] | PLoS ONE | 2015 | Caucasian | Colorectal cancer | 915 | 1719 | 839 | 76 | 0 | 1577 | 141 | 1 | 0.958 | 0.042 | 0.958 | 0.042 |
| **Winchester DA[60]** | **Prostate** | **2015** | **Caucasian** | **Prostate cancer** | **867** | **830** | **768** | **94** | **5** | **741** | **82** | **7** | **0.940** | **0.060** | **0.942** | **0.058** |
| **Zidi S[61]** | **Pathol Oncol Res** | **2016** | **African** | **Cervical cancer** | **130** | **260** | **116** | **6** | **8** | **207** | **46** | **7** | **0.915** | **0.085** | **0.885** | **0.115** |
| Semlali A[23] | PLoS ONE | 2016 | Caucasian | Colorectal cancer | 114 | 100 | 106 | 7 | 1 | 92 | 7 | 1 | 0.961 | 0.039 | 0.955 | 0.045 |
| Danyelle A Winchester[62] | Prostate | 2017 | Caucasian | Prostate cancer | 619 | 527 | 555 | 64 | 0 | 465 | 58 | 4 | 0.948 | 0.052 | 0.937 | 0.063 |
| **rs4986791** |  |  |  |  |  |  | **CC** | **CT** | **TT** | **CC** | **CT** | **TT** | **C** | **T** | **C** | **T** |
| Boraska Jelavic T[46] | Clin Genet | 2006 | Caucasian | Colorectal cancer | 89 | 87 | 77 | 12 | 0 | 82 | 5 | 0 | 0.933 | 0.067 | 0.971 | 0.029 |
| Garza-Gonzalez E[63] | BMC Cancer | 2007 | Mixed | Gastric cancer | 78 | 189 | 77 | 1 | 0 | 179 | 10 | 0 | 0.994 | 0.006 | 0.974 | 0.026 |
| Santini D[49] | Clin Exp Immunol | 2008 | Caucasian | Gastric cancer | 171 | 151 | 155 | 15 | 1 | 147 | 4 | 0 | 0.950 | 0.050 | 0.987 | 0.013 |
| Trejo-de la OA[64] | Clin Immunol | 2008 | Mixed | Gastric cancer | 61 | 202 | 57 | 4 | 0 | 193 | 9 | 0 | 0.967 | 0.033 | 0.978 | 0.022 |
| Pandey S[50] | Gynecol Oncol | 2009 | Caucasian | Cervical cancer | 150 | 150 | 127 | 21 | 2 | 133 | 16 | 1 | 0.917 | 0.083 | 0.940 | 0.060 |
| Srivastava K[27] | Liver Int | 2010 | Caucasian | Gallbladder cancer | 232 | 257 | 195 | 32 | 5 | 232 | 24 | 1 | 0.909 | 0.091 | 0.949 | 0.051 |
| Balistreri CR[53] | Curr Pharm Des | 2010 | Caucasian | Prostate cancer | 50 | 125 | 48 | 2 | 0 | 118 | 7 | 0 | 0.980 | 0.020 | 0.972 | 0.028 |
| Rigoli L[65] | Anticancer Res | 2010 | Caucasian | Gastric cancer | 70 | 87 | 57 | 13 | 0 | 81 | 6 | 0 | 0.907 | 0.093 | 0.966 | 0.034 |
| **Davoodi H[66]** | **Iran J Allergy Asthma Immunol** | **2011** | **Asian** | **Colorectal cancer** | **60** | **50** | **58** | **2** | **0** | **50** | **0** | **0** | **0.983** | **0.017** | **1.000** | **0.000** |
| Singh V[67] | Arch Med Res | 2013 | Asian | Bladder cancer | 200 | 200 | 163 | 35 | 2 | 173 | 26 | 1 | 0.903 | 0.098 | 0.930 | 0.070 |
| Theodoropoulos GE[12] | Breast | 2012 | Caucasian | Breast cancer | 261 | 480 | 253 | 8 | 0 | 466 | 14 | 0 | 0.985 | 0.015 | 0.985 | 0.015 |
| **Continued** |  |  |  |  |  |  |  |  |  |  |  |  |  |  |  |  |

| **Polymorphisms and study** | **Journal** | **Year** | **Ethnicity** | **Cancer type** | **Sample size** | | **Genotypes** | | | | | | **Allele frequencies(%)** | | | |
| --- | --- | --- | --- | --- | --- | --- | --- | --- | --- | --- | --- | --- | --- | --- | --- | --- |
|  |  |  |  |  | **Cases** | **Controls** | **Cases** | | | **Controls** | | | **Cases** | | **Controls** | |
| de Oliveira JG[68] | World J Gastroenterol | 2012 | Caucasian | Gastric cancer | 174 | 225 | 165 | 9 | 0 | 219 | 6 | 0 | 0.974 | 0.026 | 0.987 | 0.013 |
| Yang ZH[55] | Cancer Sci | 2012 | Asian | Nasopharyngeal cancer | 236 | 287 | 188 | 45 | 3 | 254 | 32 | 1 | 0.892 | 0.108 | 0.941 | 0.059 |
| Agundez JA[29] | Oncology | 2012 | Caucasian | Hepatocellular cancer | 155 | 390 | 143 | 12 | 0 | 341 | 47 | 2 | 0.961 | 0.039 | 0.935 | 0.065 |
| Dai Q[56] | Chin J Pathophysiol | 2012 | Asian | Colorectal cancer | 268 | 268 | 182 | 78 | 8 | 214 | 52 | 2 | 0.825 | 0.175 | 0.896 | 0.104 |
| **Priyadarshini A[20]** | **India J Urol** | **2013** | **Caucasian** | **Prostate cancer** | **198** | **200** | **158** | **32** | **8** | **157** | **37** | **6** | **0.879** | **0.121** | **0.878** | **0.123** |
| de Oliveira JG[39] | Dig Dis Sci | 2013 | Caucasian | Gastric cancer | 200 | 240 | 191 | 9 | 0 | 234 | 6 | 0 | 0.978 | 0.023 | 0.988 | 0.013 |
| Yang CX[11] | Tissue Antigens | 2013 | Asian | Breast cancer | 202 | 202 | 202 | 0 | 0 | 201 | 1 | 0 | 1.000 | 0.000 | 0.998 | 0.002 |
| **Shen Y[40]** | **Pathol Oncol Res** | **2013** | **Asian** | **Bladder cancer** | **436** | **522** | **433** | **1** | **2** | **517** | **3** | **2** | **0.994** | **0.006** | **0.993** | **0.007** |
| Kutikhin AG[69] | Tumor Biol | 2014 | Caucasian | Gastric cancer | 66 | 300 | 55 | 11 | 0 | 255 | 45 | 0 | 0.917 | 0.083 | 0.925 | 0.075 |
| Kutikhin AG[69] | Tumor Biol | 2014 | Caucasian | Rectal cancer | 125 | 300 | 100 | 23 | 2 | 255 | 45 | 0 | 0.892 | 0.108 | 0.925 | 0.075 |
| Kutikhin AG[69] | Tumor Biol | 2014 | Caucasian | Colorectal cancer | 233 | 300 | 195 | 36 | 2 | 255 | 45 | 0 | 0.914 | 0.086 | 0.925 | 0.075 |
| Kutikhin AG[69] | Tumor Biol | 2014 | Caucasian | Ovarian cancer | 79 | 168 | 69 | 9 | 1 | 144 | 24 | 0 | 0.930 | 0.070 | 0.929 | 0.071 |
| Companioni O[58] | Int J Cancer | 2014 | Caucasian | Gastric cancer | 354 | 1263 | 309 | 45 | 0 | 1124 | 134 | 5 | 0.936 | 0.064 | 0.943 | 0.057 |
| Omrane I[26] | Tumor Biol | 2014 | African | Colorectal cancer | 100 | 140 | 94 | 6 | 0 | 123 | 17 | 0 | 0.970 | 0.030 | 0.939 | 0.061 |
| Zeljic K[70] | Oral Dis | 2014 | Caucasian | Oral cance | 93 | 104 | 77 | 16 | 0 | 90 | 13 | 1 | 0.914 | 0.086 | 0.928 | 0.072 |
| Qadri Q[38] | Immunol Invest | 2014 | Caucasian | Gastric cancer | 130 | 200 | 114 | 16 | 0 | 182 | 18 | 0 | 0.938 | 0.062 | 0.955 | 0.045 |
| Kurt H[15] | Inflammation | 2016 | Caucasian | Lung Cancer | 160 | 100 | 156 | 4 | 0 | 91 | 9 | 0 | 0.988 | 0.013 | 0.955 | 0.045 |
| Rybka J[41] | Int J Immunogenet | 2016 | Caucasian | Acute myeloid leukaemia | 59 | 122 | 52 | 6 | 1 | 104 | 18 | 0 | 0.932 | 0.068 | 0.926 | 0.074 |
| Jin Y[44] | Mamm Genome | 2017 | Asian | Cervical cancer | 420 | 842 | 237 | 147 | 36 | 535 | 262 | 45 | 0.739 | 0.261 | 0.791 | 0.209 |
| **rs11536889** |  |  |  |  |  |  | **GG** | **GC** | **CC** | **GG** | **GC** | **CC** | **G** | **C** | **G** | **C** |
| Zheng SL[16] | Cancer Res | 2004 | Caucasian | Prostate cancer | 1380 | 778 | 1047 | 318 | 15 | 625 | 141 | 12 | 0.874 | 0.126 | 0.894 | 0.106 |
| Chen YC[45] | Cancer Res | 2005 | Caucasian | Prostate cancer | 692 | 687 | 515 | 167 | 10 | 513 | 159 | 15 | 0.865 | 0.135 | 0.862 | 0.138 |
| **Continued** |  |  |  |  |  |  |  |  |  |  |  |  |  |  |  |  |

| **Polymorphisms and study** | **Journal** | **Year** | **Ethnicity** | **Cancer type** | **Sample size** | | **Genotypes** | | | | | | **Allele frequencies(%)** | | | |
| --- | --- | --- | --- | --- | --- | --- | --- | --- | --- | --- | --- | --- | --- | --- | --- | --- |
|  |  |  |  |  | **Cases** | **Controls** | **Cases** | | | **Controls** | | | **Cases** | | **Controls** | |
| **Cheng I[21]** | **Cancer Epidemiol  Biomarkers Prev** | **2007** | **Caucasian** | **Prostate cancer** | **506** | **506** | **385** | **105** | **16** | **401** | **93** | **12** | **0.865** | **0.135** | **0.884** | **0.116** |
| Wang MH[17] | Prostate | 2009 | Caucasian | Prostate cancer | 254 | 252 | 178 | 69 | 7 | 175 | 71 | 6 | 0.837 | 0.163 | 0.835 | 0.165 |
| Hishida A[71] | Helicobacter | 2009 | Asian | Gastric cancer | 583 | 1592 | 312 | 222 | 49 | 827 | 635 | 130 | 0.726 | 0.274 | 0.719 | 0.281 |
| Kupcinskas J[72] | BMC Med Genet | 2011 | Caucasian | Gastric cancer | 113 | 236 | 90 | 21 | 2 | 190 | 41 | 5 | 0.889 | 0.111 | 0.892 | 0.108 |
| Minmin S[28] | PLoS One | 2011 | Asian | Hepatocellular cancer | 216 | 228 | 123 | 76 | 17 | 123 | 91 | 14 | 0.745 | 0.255 | 0.739 | 0.261 |
| Shui IM[19] | Prostate | 2012 | Caucasian | Prostate cancer | 1233 | 1215 | 909 | 292 | 32 | 897 | 291 | 27 | 0.856 | 0.144 | 0.858 | 0.142 |
| He C[73] | Carcinogenesis | 2013 | Asian | Gastric cancer | 231 | 539 | 146 | 73 | 12 | 343 | 175 | 21 | 0.790 | 0.210 | 0.799 | 0.201 |
| Zhu L1[22] | Med Oncol | 2014 | Asian | Colon cancer | 214 | 627 | 115 | 81 | 18 | 345 | 245 | 37 | 0.727 | 0.273 | 0.746 | 0.254 |
| Zhu L2[22] | Med Oncol | 2014 | Asian | Rectal cancer | 387 | 627 | 191 | 171 | 25 | 345 | 245 | 37 | 0.714 | 0.286 | 0.746 | 0.254 |
| Companioni[58] | Int J Cancer | 2014 | Caucasian | Gastric cancer | 365 | 1283 | 258 | 98 | 9 | 940 | 308 | 35 | 0.841 | 0.159 | 0.853 | 0.147 |
| Li P[74] | Genet Test  Mol Biomarkers | 2014 | Asian | Gastric cancer | 409 | 409 | 331 | 74 | 4 | 328 | 77 | 4 | 0.900 | 0.100 | 0.896 | 0.104 |
| Castano-Rodriguez N[43] | Hum Immunol | 2014 | Asian | Gastric cancer | 85 | 212 | 43 | 33 | 9 | 131 | 74 | 7 | 0.700 | 0.300 | 0.792 | 0.208 |
| Jin Y[44] | Mamm Genome | 2017 | Asian | cervical cancer | 424 | 842 | 221 | 159 | 44 | 486 | 299 | 57 | 0.709 | 0.291 | 0.755 | 0.245 |
